# A comparative study on the blood and milk cell counts of healthy, subclinical, and clinical mastitis Karan Fries cows

**DOI:** 10.14202/vetworld.2015.685-689

**Published:** 2015-05-29

**Authors:** Mohanned Alhussien, Mandheer Kaur, Pasumarti Manjari, Shiv Prasad Kimothi, Ashok K. Mohanty, Ajay K. Dang

**Affiliations:** Division of Dairy Cattle Physiology, Lactation and Immunophysiology Laboratory, National Dairy Research Institute, Karnal - 132 001, Haryana, India

**Keywords:** blood, milk, cell counts, healthy, mastitis, cows

## Abstract

**Aim:**

The present study was aimed to study the use of cell counts as an early indicator of mammary health.

**Materials and Methods:**

Milk and blood cell counts were estimated from 8 healthy, 8 subclinical (SCM), and 8 clinically mastitis (CM) groups of Karan Fries (KF) cows.

**Results:**

Total leucocyte counts and neutrophil percent in blood and milk somatic cells and milk neutrophil percent of healthy cows increased significantly (p<0.05) in SCM cows and CM cows. Viability of blood and milk neutrophils was more in healthy cows, but decreased significantly (p<0.05) in SCM and CM cows. Significant (p<0.05) decrease were also observed in both the blood and milk lymphocytes and monocytes of SCM and CM cows. Phagocytic activity (PA) of blood neutrophils also decreased significantly (p<0.05) in SCM cows. There was no difference between the PA of SCM and CM cows. Milk neutrophil percent was more in the SCM and clinically infected milk than in the blood of these cows. About 96-97% of the neutrophils had segmented nucleus in both healthy and subclinical milk, whereas, 2-3% were having band shaped or immature nuclei. There was a significant decrease in the segmented neutrophils, whereas, band neutrophils increase significantly to about 5% in the infected milk of mastitic cows. Viability of the milk neutrophils decreased more in case of subclinical and clinical milk as compared to that of blood. PA was found to be highest in the milk of healthy group of cows, but decreased significantly (p<0.05) in subclinically infected cows. However, there was no difference between the PA of milk neutrophils of SCM and CM cows. PA of milk was also found to be significantly lower in the milk of healthy cows when compared to that of blood neutrophils.

**Conclusion:**

This study indicated that percent neutrophils and their type in conjunction with milk somatic cell counts can be used as a more reliable indicator of mammary health in cows.

## Introduction

Mastitis is considered as one of the costliest diseases of dairy animals. Mastitis occurs when a pathogen enters the mammary gland of a cow and tries to establish itself within the gland. Subclinical form of mastitis was found more important in India (varying from 10% to 50% in cows and 5-20% in buffaloes) than clinical mastitis (1-10%). The incidence was highest in Purebred Holsteins and Jerseys and lowest in local cattle and buffaloes [1]. Mastitis whether subclinical or clinical leads to an increase in milk cells, particularly the milk leucocytes, decrease in the milk producing mammary epithelial cells, and an altered milk composition.

Neutrophils are the most vital primary mobile phagocytes [2] in the body of mammals and play a key role in initiating an innate, inflammatory, and specific immune response. With the invasion of pathogens, the neutrophils migrate from the blood into milk and employ cascades of reactions including both oxidative and non-oxidative mechanisms to destroy the pathogens. Several studies have been carried out on the incidence and treatment of mastitis, but the complex nature of pathogenesis and immune responses in mastitis are still poorly understood [3].

Therefore, the present study was undertaken to see what changes occur in the cellular composition of cows suffering from SCM or CM both in blood, as well as milk. Further we can take a particular cell as a marker for early detection of SCM.

## Materials and Methods

### Ethical approval

The present study was undertaken after getting necessary approval from the Institute’s Animal Ethics Committee.

### Selection of animals

The present study was conducted in Karan Fries (KF) cows which are a cross between indigenous Tharparkar and Holstein Friesian cows. The average age at calving is 30-32 months and the milk production is around 3700 kg with 3.8-4.0% fat. The inter calving period is 400-430 days. 24, KF cows, in their early lactation (30-60 days) were selected for the study. All the cows were multiparous having 3-6 parity. These cows were divided into 3 groups *viz*.; healthy (n=8), subclinical (n=8), and clinical mastitis (n=8) cows by estimating milk somatic cell counts (SCC) and California Mastitic test in them. The cows were managed as per the practices followed in the institute’s herd. They were offered *ad lib* green fodder and calculated amount of concentrate mixture. Fresh tap water was available *ad lib* at all the time of the day.

### Blood and milk sampling

Blood samples were collected from all KF cows in vacutainers and analyzed for total leucocyte counts (TLC), differential leucocyte counts (DLC), type of neutrophils, immature having band nuclei, and mature having segmented nuclei. Milk samples were collected fresh and brought to the laboratory for further analysis.

### Analysis

Viability of blood neutrophils was estimated by Trypan blue exclusion method. This method is based on the principle that since the dead cells take up the dye they appear blue whereas the live cells appear colorless since they do not take up the dye. Blood TLC was measured by counting the white blood cells on a hemocytometer under a microscope. For estimating blood DLC, a blood smear was prepared on a clean glass slide. The stained blood smear was examined under an oil immersion objective for accurate cell identification. All the leucocytes were classified. About 100 cells were counted to determine the % of different leucocytes.

All materials and reagents used for the isolation of blood and milk polymorphonuclear neutrophils (PMN) were sterile. Isolation of PMN from peripheral blood was performed using hypotonic lysis of erythrocytes [4]. Briefly, 10 ml of ethylenediaminetetraacetic acid mixed blood was poured into the Falcon tubes and centrifuged (1000 × g, 15 min., 4°C); the plasma layer, buffy coat, and top layer of the blood-packed cells were discarded. About 2.5 ml of the blood-packed cell was lysed by adding 5 ml of double distilled water and gently mixed for 45 s. usinga magnetic stirrer. After restoration of the isotonicity by addition of 2.5 ml of 2.7% NaCl with gentle mixing for 60 s., the suspension was centrifuged (1000 × *g*, 10 min., and 4°C). For the second lysis procedure, after resuspending of the pellets in 2.5 ml of Dulbecco’s phosphate buffered saline (PBS) (without CaCl_2_ and MgCl_2_), 5 ml of double distilled water was added and gently mixed for 30 s, then 2.5 ml of 2.7% NaCl was added, gently mixed for 60 s, and centrifuged (1000 × *g*, 5 min., 4°C). The remaining cell pellet was washed 3 times in PBS (300 × *g*, 10 min., 4°C) and the final cell pellet was resuspended in RPMI media for further analysis.

Estimation of phagocytic activity (PA) was based on the principle that phagocytic cells produce O_2_^−^ anions. These anions reduce the yellow colored water-soluble nitroblue tetrazolium (NBT) to water insoluble blue or purple colored formazan crystals. The OD of the reduction product was determined at 540 nm [5]. For estimating PA, 1 million viable cells were taken in each well of 96 well culture plates. Two different volume of the same concentration of zymosan and NBT were tried. Five treatment well of ELISA plate contained 10 µl NBT along with 10 µl zymosan and cells. Five other treatments well contained 20 µl NBT along with 20 µl zymosan and cells. Five blank well contained only cells. Their final volume was adjusted to 200 µl by adding media. It was incubated for 2 h in CO_2_ incubator at 5% CO_2_ and 95% air level. OD was taken at 540 nm. Same method was repeated for estimating PA of milk neutrophils.

SCC of milk samples of all the 3 groups were measured by a somatic cell counter and also cross checked by making a milk smear and observing the milk smear glass slide under a microscope at ×40. Differential cell counting was also carried out to determine the presence of different cell types like lymphocytes, neutrophils, and macrophages in milk [6]. SCC of each original milk sample was determined in duplicate within 6 h post collection. The milk was heated to 40°C in a water bath held for 15 min at that temperature before being cooled to 20°C with careful stirring. 0.01 ml of milk was spread on a 1 cm^2^ (0.5 cm × 2 cm) area of a degreased microscopic slide and was dried in a horizontal position. After drying overnight, duplicate smears were fixed with 96% ethyl alcohol (3 min), air dried, defatted with xylol (8 min), and rinsed smoothly with 60% ethyl alcohol, air dried, successively dyed with pure May-Grünwald (2 min), at 50% (2 min) and Giemsa solution (20 min), air dried, and dehydrated in an increasing series of alcohols and xylols.

### Statistical analysis

Statistical analysis was performed as per the standard procedure. Statistical analyses were performed using the SPSS 17.0 statistical software package. The means were compared using Analysis of Variance and presented as mean ± standard error. The minimum significant range of confidence was evaluated at 0.05 level.

## Results

Milk and blood cell counts were estimated from healthy, subclinical, and clinically mastitis group of KF cows. Results of blood DLC, type of neutrophils, their viability, and PA have been presented in Table-1.

**Table-1:** Blood TLC, differential leukocyte counts, and phagocytic activity of blood neutrophils in healthy, subclinical, and clinically infected KF cows.

Blood	Healthy	Subclinical mastitis	Clinical mastitis
TLC (×10^3^)	7293±35.52^a^	8417±55.96^b^	9237±95.55^c^
Neutrophil (%)	24.66±0.28^a^	38.78±0.34^b^	60.01±0.70^c^
Segmented n (%)	97.5±0.46	97.25±0.49	96.88±0.54
Band n (%)	2.5±0.46	2.75±0.49	3.125±0.54
Viability n (%)	93.33±0.31^a^	84.42±0.51^b^	80.78±0.60^c^
Lymphocyte (%)	57.29±0.27^a^	48.29±0.22^b^	19.44±0.46^c^
Monocyte (%)	6.23±0.17^a^	3.98±0.07^b^	5.42±0.20^c^
PA	0.54±0.02^a^	0.25±0.01^b^	0.30±0.01^b^

The figures bearing different alphabets differ significantly at p<0.05, PA=Phagocytic activity, TLC=Total leukocyte counts, KF=Karan Fries

### Number and PA of blood cells

TLCs in the blood of healthy cows were 7293±35.52 per µl of blood, which increased significantly (p<0.05) to 8417±55.96 in SCM cows, followed by 9237±95.55 per µl of blood in clinically infected cows. A significant increase was also observed in the blood neutrophil percentage of subclinical and clinical mastitic cows. On observing the type of neutrophils under the microscope, about 97% of the neutrophils showed segmented nucleus, whereas, 2-3% were having band shaped or immature nuclei in healthy cows, SCM and CM cows. Viability of the blood neutrophils showed that viability was 93% in healthy cows, but this viability decreased to 84 and 80%, respectively, in subclinical and clinical mastitic cows. A significant (p<0.05) decrease were also observed in blood lymphocytes and monocytes of SCM and CM cows. PA was found to be highest in the healthy group of cows. This PA decreased significantly (p<0.05) in subclinically infected cows. There was no difference between the PA of SCM and CM cows.

### Number and PA of milk cells

**Figure-1:**
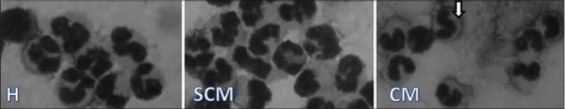
Milk neutrophils in healthy, subclinical, and mastitis Karan Fries cows (¯ indicates immature neutrophils).

The results of milk DLC, type of neutrophils, their viability and PA have been presented in Table-2. Total SCC in milk of healthy cows were 2.55±0.23 lakh per ml of milk, which increased significantly (p<0.05) to 5.97±0.28 in SCM cows and then again to 12.19 lakh ± 0.56 per ml in the milk of clinically infected cows. A significant increase was also observed in the milk neutrophil percentage of subclinical and clinical mastitic cows. On comparing the neutrophil percentage between milk and blood, milk neutrophil percent was more in the subclinically and clinically infected milk than in the blood of these cows. It was seen that about 96-97% of the neutrophils had segmented nucleus in both healthy and subclinical milk, whereas, 2-3% were having band shaped or immature nuclei. There was a significant decrease in the segmented neutrophils, whereas, band neutrophils increase significantly to about 5% in the infected milk of mastitic cows (Figure-1). Viability of the milk neutrophils decreased more in case of subclinical and clinical milk as compared to that of blood. A significant (p<0.05) decrease were also observed in milk lymphocytes and monocytes of SCM and CM cows as also observed in blood samples of these cows. PA was found to be highest in the healthy group of cows. This PA decreased significantly (p<0.05) in subclinically infected cows. There was no difference between the PA of SCM and CM cows. PA of milk was also found to be significantly lower in the milk of healthy cows when compared to that of blood neutrophils.

**Table-2:** Milk SCC, differential leukocyte counts, phagocytic activity of milk neutrophils, healthy, subclinical, and clinically infected KF cows.

Milk (%)	Healthy	Subclinical mastitis	Clinical mastitis
SCC (×10^5^)	2.55±0.23^a^	5.97±0.28^b^	12.19±0.56^c^
Neutrophil (%)	18.5±0.45^a^	43.29±0.58^b^	77.4±0.59^c^
Segmented n (%)	97.13±0.44^a^	96.63±0.56^a^	94.13±0.47^b^
Band n (%)	2.62±0.41^a^	3.12±0.44^a^	5.87±0.47^b^
Viability n (%)	91.53±0.42^a^	82.77±0.60^b^	70.78±0.87^c^
Lymphocyte (%)	14.58±0.48^a^	11.58±0.36^b^	5.6±0.25^c^
Macrophage (%)	66.92±0.68^a^	45.13±0.62^b^	17.00±0.63^c^
PA	0.45±0.01^a^	0.37±0.01^b^	0.34±0.01^b^

The figures bearing different alphabets differ significantly at p<0.05, PA=Phagocytic activity, KF=Karan Fries, SCC=Somatic cell counts

## Discussion

This study carried out on KF cows demonstrated some differences in the immune cells of peripheral blood and milk among cows with different mammary health conditions. TLC is a blood test that measures the number of white blood cells in an animal and both increased and decreased values suggest some underlying abnormality in that animal. TLC observed in this study was within range as observed in normal cows [7]. TLC increased in subclinical and clinical cases of mastitis due to invasion by a pathogen inside the mammary gland. An increase in erythrocyte sedimentation rate and white blood cells in the blood of mastitic cows has also been observed by some workers [8]. However, these results were different to that obtained in Murrah buffaloes in our earlier studies [9] where we could not found any change in the TLC of mastitic buffaloes, which indicated that the condition might be localized affecting only the udder without systemic involvement. Values of DLC in blood also changed indicating the level of stress in the body, but this could not be compared due to lack of literature on this aspect in mastitis cows.

The production of high-quality milk is a requirement to sustain a profitable dairy industry and SCC values are routinely used to identify subclinical mastitis and define quality standards in developed countries [10]. Somatic cells of milk are white blood cells and epithelial cells, which slough off from the lining of the mammary gland during the normal course of milking [11]. They are widely used as marker to determine the mammary health and quality of milk [12,13]. Milk from healthy mammary gland has about 10^5^ somatic cells/ml and values higher than this indicates secretary disturbances [6]. About 270,000 cells/ml of milk SCC have been reported in healthy Austrian Bălțat with Red Holstein cows [14]. Milk SCC increase significantly in mastitic cows and act as a protective mechanism to kill the invading pathogens in the mammary gland. An increase in the severity of mastitis leads to a significant increase in milk SCC in buffaloes [15]. This increment in SCC not only deteriorates udder health, but also affects milk quality by releasing high amount of protease and lipase enzymes, which are highly heat resistant in nature and cause problem during processing of milk and milk products [16]. Milk SCC found in healthy KF cows in our study were lower than those reported in Holstein Friesian and Brown Swiss cows [17]. This is because the milk yield of KF cows is significantly lesser than exotic cows and also SCC is positively correlated with milk yield.

Of all the cells, we observed a maximum increase in the milk neutrophils in our study. Neutrophils are the first cells to migrate from the blood into an inflamed area after initiation of inflammation. In the infected quarter of subclinical and clinical KF cows, the neutrophils increased to 43 and 77%, respectively, which was <90% as reported in exotic cows [16]. This influx of neutrophils in infected quarters was 4 times more than that found in the milk of normal quarters. Our study also substantiates already existing information that the maximum disruption of the integrity of the alveolus occurs during mastitis. This step is essential not only to supply leucocytes to fight invading pathogens but also to provide the passage of immune components to reach the site of infection. Blood neutrophils also increased two times more in clinical cows, but their amount was lesser than that observed in milk, indicating that milk neutrophils exhibited a sharp increase in their values in the infected quarters. We also observed more immature, band-shaped neutrophils only in the milk of infected cows. This occurs because as more and more neutrophils have to be sent to the mammary gland to fight an infection, bone marrow releases more immature neutrophils. These neutrophils reach the mammary gland but cannot exhibit their full potential due to their immaturity.

The main function of neutrophils is phagocytosis and intracellular killing by engulfing bacteria by two distinct mechanisms, the respiratory burst, and by digestion by lysosomal enzymes. An increase in blood neutrophils in high producing cows may be due to stress on the mammary gland to produce more milk. The ability of neutrophils to phagocytose foreign particles is important for protection of the mammary gland and *in vitro* analysis of neutrophil function provides a very effective tool for the study of natural mastitis resistance [18]. PA and index were found to be lower in milk than in blood neutrophils. The reason for this is that as milk neutrophils have reduced glycogen stores compared to that of blood, this may limit the availability of energy. Milk neutrophils also have reduced abilities to produce reactive oxygen species, as compared to that of blood [19]. The decreased bactericidal activity of milk PMN in comparison with blood PMN could also be explained by a lower superoxide production measured by chemiluminescence and a lower H_2_O_2_ production measured by flow cytometry [20]. PA of milk leukocytes was lower in mastitic cows, whereas, a significant enhancement in PA was recorded in post-treated cows [21].

The current study was conceived with the goal of understanding the biological function of the leucocytes when they are transported from the blood circulation into milk to provide cellular immunity during early lactation. Period of early lactation was taken in this study as early lactation is the most critical period because during this period the mammary gland undergoes marked biochemical, cellular and immunomodulatory changes to synthesize colostrum, transform colostrum into milk, and then for the attainment of peak milk production. All these transformations put pressure on the mammary gland and increase the chance of new intramammary infections during this time.

## Conclusion

This study provides comparative information on the changes in the blood and milk cell counts occurring in healthy, subclinical, and clinical mastitis KF cows. This study also indicates that estimation of milk neutrophils along with their type (mature or immature) along with milk SCC is a better indicator of the milk quality and udder health than milk SCC alone. Because milk SCC can be high or low depending upon the milk yield, environmental conditions, feeding and genetic makeup of the animal (indigenous cows have low SCC as compared to exotic cows). Whereas, concentration, type, and activity of neutrophil changes only when there is any infection or change in the immune status of the mammary gland. Therefore, by quantifying milk neutrophils and SCC together we can get a clearer picture of the stress faced by the mammary gland.

## Authors’ Contributions

MA, SPK, AKM, and AKD designed the entire study. MK and PM helped in doing the SCC and staining of cells. PM also brought the samples from the cattle yard to Lab. MK isolated the cells from blood and milk. MA carried out all experiments along with analysis of data. AKD finalized the manuscript for communication to the journal. All the authors read the manuscript and approved the final manuscript.
